# Implementing the Family Talk Intervention among families with a severely ill parent or child with palliative care needs- a longitudinal study of the perspectives of hospital social workers

**DOI:** 10.3389/frhs.2025.1527431

**Published:** 2025-06-13

**Authors:** Ingrid Thermaenius, Maja Holm, Kristofer Årestedt, Camilla Udo, Anette Alvariza, Tina Lundberg, Lars Wallin, Malin Lövgren

**Affiliations:** ^1^ Department of Health Care Sciences, Marie Cederschiöld University, Stockholm, Sweden; ^2^ Department of Nursing Sciences, Sophiahemmet University, Stockholm, Sweden; ^3^ Department of Health and Caring Sciences, Faculty of Health and Life Science, Linnaeus University, Växjö, Sweden; ^4^ School of Health and Welfare, Dalarna University, Falun, Sweden; ^5^ Research and Development/Palliative Care, Stockholms Sjukhem, Stockholm, Sweden; ^6^ Medical Unit: Clinical Social Work, Karolinska University Hospital, Stockholm, Sweden; ^7^ Advanced Pediatric Home Care, Astrid Lindgren Children’s Hospital, Karolinska University Hospital, Stockholm, Sweden

**Keywords:** implementation, normalization process theory, psychosocial support, hospital social worker, family based intervention

## Abstract

**Background:**

The Family Talk Intervention (FTI) is a psychosocial intervention supporting families where a family member has palliative care needs. This study aimed to evaluate how the Family Talk Intervention (FTI) was implemented over time from the perspective of hospital social workers (HSWs) in their everyday clinical practice among families with a severely ill parent or child in need of palliative care.

**Methods:**

HSWs (*n* = 21) working in adult and children's care completed a 10-day education where they were trained to use FTI. The education was part of a multifaced implementation strategy involving educational outreach visits, facilitation, clinical implementation meetings, and audit and feedback. The HSWs were then expected to use FTI in their clinical practice to support families with dependent children. To assess if and how FTI was integrated into their daily practice, they were also asked to complete the Swedish version of the Normalization Process Theory Measure (S-NoMAD) on three occasions: on completion of the FTI-education, six months later, and one year later. For the longitudinal analysis of data, Friedman's test was used.

**Results:**

The HSWs rated the use of FTI high after completing the FTI-education, indicating a positive attitude towards FTI. In the longitudinal analysis, statistically significant changes were seen for two questions in S-NoMAD, where the HSWs' ratings showed that the FTI became more familiar and normalized over time. Generally, the HSWs' ratings of S-NoMAD's main constructs were high and stable over time, indicating a positive view of FTI and its implementation. However, for the single questions, the ratings were slightly more negative to some contextual aspects, such as managerial support and resources.

**Conclusion:**

As results showed, HSW mainly rated different aspects of the implementation process as positive, both from the beginning, but also over time. Therefore, the intervention could be judged to have been implemented as a tool to support families when a parent or a child is severely ill. Contextual factors, involving managerial support and resources were rated lower, indicating the importance of those aspects when introducing interventions into healthcare. The result also indicates that the multifaced implementation strategy supported the HSW's everyday clinical practice.

**Clinical Trial Registration:**

clinicaltrials, nr, identifier (NCT05365919; 2022-03-04 and; NCT05020158 2021-05-11).

## Introduction

Regardless of whether the ill person is a parent or a child, being severely ill and having palliative care needs affects all family members but can be particularly stressful in a family with dependent children (1–3). Hence, family members commonly need psychosocial support, both as individuals and as a group (4, 5). Children in these families are often in need of psychosocial support involving illness-related information (6), and parents need support in how to talk to their children (7, 8). Hospital social workers (HSWs), also referred to in research publications as medical social workers, healthcare social workers or healthcare counselors, have an important role in healthcare in providing these families with psychosocial support (9–11). Psychosocial support is based on a holistic view of the patient, including psychological, physical, social, and existential factors (12, 13). To provide appropriate psychosocial support to families affected by severe illness, HSWs need access to evidence-based psychosocial interventions; however earlier review studies have stated that there is a shortage of family-targeted interventions with proven effect when a family member has a severe illness (14–16). Even fewer of the interventions involve the entire family. Due to the shortage of such interventions, there is a lack of knowledge regarding how to best implement them within healthcare (17–19). Previous research shows that successful implementation of psychosocial interventions for families affected by severe illness is closely linked to the intervention being integrated into routine care and having a flexible structure (20, 21). Barriers includes lack of resources, and that the intervention not being sufficiently tailored to the families' unique situation.

One family-based psychosocial intervention that has been pilot-tested in families with dependent children affected by severe illness is The Family Talk Intervention (FTI), with positive results regarding both feasibility and potential effects (22–25). However, when an intervention is tested in real health care contexts, aspects of the implementation process must also be evaluated to understand why the intervention works (26). Within implementation science there are several theoretical frameworks that can be used to plan and evaluate an implementation process (27). One of those theoretical frameworks is *Normalization Process Theory* (NPT), which aims to define and clarify factors that may influence the implementation of new interventions within healthcare (28, 29). Normalization is a key concept, used to explore and assess how health care professionals and team works to integrate new interventions into routine work (30).

Various factors, such as healthcare professionals' responses to the intervention, implementation climate and culture, and context will affect the process of new interventions becoming a routine in healthcare (31–33). In studies of complex interventions such as FTI, which includes several different components, reports on the implementation process of the interventions have been few, with a lack of homogenous definitions and descriptions of the implementation strategies used and healthcare professionals' responses (34–36).

Studying aspects of the implementation process could potentially lead to an increased understanding of factors that are of importance in making a psychosocial intervention such as FTI become routine (30, 37, 38).

## Material & methods

### Aim

The aim of this study was to evaluate how the Family Talk Intervention (FTI) was implemented over time from the perspective of HSWs in their everyday clinical practice among families with a severely ill parent or child in need of palliative care.

### Study design and setting

This study is a part of a larger project that was carried out between the years 2021–2024 in which FTI is evaluated with an effectiveness-implementation hybrid design, type 2 (39). The current study used the Normalization Process Theory (NPT) as the basis for the evaluation of the implementation of FTI. NPT characterizes and explains key mechanisms that promote and inhibit the implementation, embedding, and integration of complex interventions in healthcare (28, 29).

The study has a longitudinal design based on quantitative data collected over a year from 19 HSWs who were educated and trained in using FTI. In addition to the HSWs, the study participants also included two nurses. However, for readability purposes, all participants (21) will be referred to as HSWs throughout the manuscript.

The HSWs all worked with families affected by severe illness in four different healthcare contexts in an urban area in Sweden: (1) Specialised palliative home care for adults, where the HSWs were included in multi-professional teams that offered 24 h care. Patients in these settings had diagnoses such as cancer, cardiovascular diseases, and chronic obstructive pulmonary diseases. (2) A university hospital with cancer care units for adults with different types of cancer, both emergency care and outpatient care. (3) A pediatric hospital, with both emergency care and outpatient care (all pediatric units, e.g., neurology, oncology, haematology, intensive care). (4) A children's hospice for pediatric palliative care.

### The family talk intervention (FTI)

FTI, also called Beardslee's family intervention, is a psychosocial family-based intervention, built on systems theory and originally developed in the context of psychiatry for families where a parent has an affective disorder (40, 41). FTI is manual-based and involves all family members. The main goals are to facilitate family communication about illness-related subjects, support parenting, increase knowledge about the illness, and emphasize the children's needs (42). For this study, the original FTI-manual was modified for the care contexts in focus (23, 43). Resilience is a key goal of FTI, since the intervention aims to help the family to identify their strengths and protective factors to help them cope with the current situation (40). FTI involves six meetings (meetings 1–6), including various family members, (with extra meetings 7–11 if needed), led by a HSW (or other healthcare professional) educated and trained in FTI (44), (Table 1). The FTI meetings are held at intervals of 1–2 weeks (45). FTI has an eclectic approach that includes psycho-educative, narrative, and dialogical ways of working (42). The psycho-educative element is focused on raising awareness about illness-related problems, such as children as next-of-kin or prognosis. FTI also includes the family's own story: individual family members share their perspectives to create a common story, the narrative element. Further, FTI has a dialogical perspective: highlighting difficult situations by emphasizing the children's voices and sharing and seeing the different perspectives of all family members.

**Table 1:** The family talk intervention: focus of each meeting and participating family members.

Meeting	Participating family members	Content
1–2	Parents/guardians	The parent's story, thoughts, and any concerns about the child/children. Parents may formulate a goal with FTI.
3	The child/The children	The child's/children's view of the illness and the situation; any questions or worries that the child has.
4	Parents/guardians	Summary of the third meeting with the child/children and preparation for meeting 5 (“the family talk”).
5	The whole family	“The family talk”, which is based on questions and worries raised at previous meetings by parents or children.
6	The whole family or the parents/guardians	Follow-up with a focus on the continued communication within the family to achieve the previously set goals for the family.
7–11	The whole family or parts of the family	If needed, extra meetings can be conducted.

### Implementation strategies

**Figure 1:**
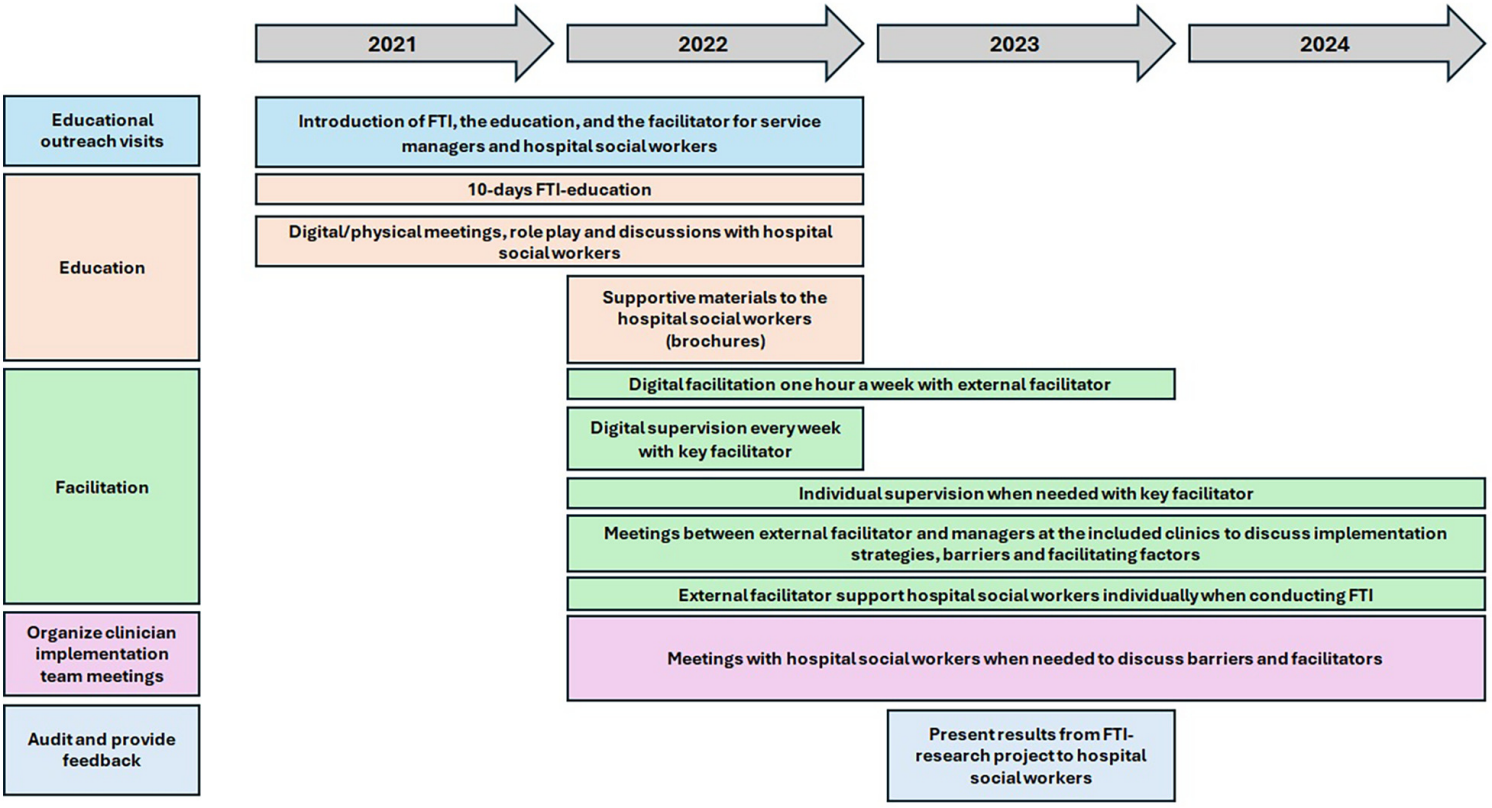
The multifaced implementaion strategy.

The implementation strategy to support the integration of FTI was multifaced, including educational outreach visits, education, facilitation, clinician implementation meetings, and audit and feedback. The components of the multifaced implementation strategy are presented in detail below and further described through the ERIC taxonomy (Expert Recommendations for Implementing Change) (36), (Figure 1).

#### Educational outreach visits

The implementation program started with *educational outreach visits* with managers and HSWs at the included clinics to present the FTI-research project and inform about the intervention, the implementation strategies, such as the FTI-education, and the role of the facilitator (see below).

#### Education

The HSWs were educated and trained in FTI for 10 days, which included practical training (e.g., role play) and supervision in relation of using the intervention in clinical practice.

The *FTI-education* consisted of 10 lectures in total held by two HSWs with special training in, and extensive experience of working with FTI. The first lecture focused on the theories included in FTI and the research conducted in this area. Lectures 2–7 included a review of the various meetings in FTI, as well as role plays to practice the intervention. Lecture 8 included discussions about the implementation of FTI into various healthcare contexts and group supervision. Group supervision was also offered in lectures 9–10. As part of the FTI-education, each HSW, together with a colleague, performed FTI under supervision with two families.

To *make the training dynamic* it varied to suit different contexts and learning styles, for example digital/physical meetings, role play and discussions. Moreover, *educational materials* were developed in the form of brochures to facilitate the process of inviting families to participate in FTI.

#### Facilitation—external facilitator

To activate the implementation process, the HSWs were provided with group support and individual support from facilitators in accordance with the Toolkit for facilitators (46). A facilitator is a person who takes on the role of providing active support to the target group (33), in this case the HSWs during the implementation process. The aim was to support the HSW in a flexible way, by understanding contextual barriers to, and facilitators of, the use of FTI. After the FTI-education, the HSWs had weekly access to a digital room where they could talk to external facilitators about the intervention. The external facilitators were members of the research group with different professional backgrounds (nurses, hospital social workers). They also had experience of palliative care research and from developing FTI in the previous pilot studies.

During their use of the intervention, every HSW conducting FTI also received individual support from an external facilitator. When needed, HSWs could get in immediate contact with an external facilitator by calling or emailing. To discuss implementation strategies, barriers and facilitating factors, meetings were held between managers and external facilitators at the included clinics when required.

#### Facilitation—key facilitator

The HSWs also had access to a key facilitator, one of the FTI-educators who had extensive experience in conducting FTI in the previous FTI pilot studies. During the implementation period, the key facilitator, employed (20% FTE) in the project, providing weekly group supervision and individual supervision when required. The key facilitator was also accessible via phone/email every day for individual supervision and support regarding the implementation of FTI. During the research project, the key facilitator also assisted some HSWs in the recruitment of families to FTI.

#### Organization of clinician implementation team meetings

During the implementation period of FTI, when required, meetings were held with the researchers and the HSWs to discuss barriers and facilitators during the implementation of FTI.

#### Audit and provide feedback

Meetings were held between the research group and the FTI-educated HSWs from all the included clinics to allow them to present their experiences of using the intervention, both from their own professional and the family's perspectives. Result from the effectiveness study were shared to support the implementation process. The aim was to learn from the use of the intervention, but also to be inspired by what FTI has given the families.

### Intervention delivery

After their FTI-education, the HSWs in various clinical settings invited families with a parent or child with a severe illness to participate in FTI. The families were included based on the definitions of palliative care for adults (47), and children (48), respectively. Further inclusion criteria were that families in adult care should have at least one child/youth aged between 0 and 24 years, and that families in pediatric care should have a child between 0 and 18 years. All families needed to be able to communicate in Swedish. During the implementation period, HSWs delivered FTI to 70–80 families affected by severe illness. FTI could be delivered in physical or virtual meetings with the families.

### Data collection

Managers in the clinical settings included consented to their units being part of the FTI- project. According to the principles of the Declaration of Helsinki (49), HSWs were informed (verbally and in writing) about the current implementation study and were asked to provide informed consent to participate. In total, 43 HSWs with permanent employment in the four different healthcare contexts consented to participate in the current study after they had taken part in FTI-education and training during 2021–2022.

To follow the implementation of FTI over time, HSWs were asked to answer web-based questionnaires at three time points: directly after the FTI-education (time point 1, (T1), after 6 months (T2), and after 12 months (T3). The questionnaires were sent to the HSWs' professional e-mail addresses with a link to the questionnaire in the web-based program Webropol. Reminders were sent if they did not respond within three weeks. Part of the questionnaire was used in the current study, which was based on the Swedish Normalization Process Theory Measure (S-NoMAD) (Table 2). The web-based questionnaires also included questions about the implementation process, such as the organizational context, as well as background characteristics, such as age, sex, and workplace.

**Table 2:** Overview of the mechanism, sub-constructs and items of NoMAD.

Mechanism	Sub-constructs	Items
Coherence	Differentiation	N4. I can see how the [intervention] differs from usual ways of working
	Communal specification	N5. Staff in this organisation have a shared understanding of the purpose of this [intervention]
	Individual specification	N6. I understand how the [intervention] affects the nature of my own work
	Internalization	N7. I can see the potential value of the [intervention] for my work
Cognitive Participation	Initiation	N8. There are key people who drive the [intervention] forward and get others involved
	Legitimation	N9. I believe that participating in the [intervention] is a legitimate part of my role
	Enrolment	N10. I am open to working with colleagues in new ways to use the [intervention]
	Activation	N11. I will continue to support the [intervention]
Collective Action	Interactional workability	N12. I can easily integrate the [intervention] into my existing work
	Relational integration	N13. The [intervention] disrupts working relationships
	Relational integration	N14. I have confidence in other people's ability to use the [intervention]
	Skill-set workability	N15. Work is assigned to those with skills appropriate to the [intervention]
	Skill-set workability	N16. Sufficient training is provided to enable staff to use the [intervention]
	Contextual integration	N17. Sufficient resources are available to support the [intervention]
	Contextual integration	N18. Management adequately supports the [intervention]
Reflexive Monitoring	Systematization	N19. I am aware of reports about the effects of the [intervention]
	Communal appraisal	N20. The staff agree that the [intervention] is worthwhile
	Individual appraisal	N21. I value the effects the [intervention] has had on my work
	Reconfiguration	N22. Feedback about the [intervention] can be used to improve it in the future
	Reconfiguration	N23. I can modify how I work with the [intervention]

### Instruments

The S-NOMAD (Normalization Measure Development) instrument is based on *The Normalization Process Theory* (NPT), translated and adjusted to Swedish conditions (28, 29, 50). The concept of normalization is central to NPT and the theory defines and clarifies factors that influence the implementation of new interventions into healthcare settings by focusing on how individual healthcare professionals and teams work to make new interventions become embedded and integrated into routine work processes (29, 51). The NPT is used within complex interventions to assess the implementation process from the perspective of the involved individuals. It is based on four constructs: *Coherence* (the sense-making work done to understand the new practice)*, Cognitive Participation* (the relational work done to engage people in the new practice)*, Collective Action* (the operational work done to enact the new practice)*,* and *Reflexive Monitoring* (the appraisal work done do to assess the new practice (29, 52), (Table 2).

S-NoMAD consists of 23 questions. The first three are general questions about the intervention that can be answered on a 10-point numeric rating scale, ranging from “not at all” (0) to “completely” (10). These questions are usually regarded as single questions (1). Further, the instrument includes 20 detailed questions about the implementation of the intervention divided over the four constructs described above (Table 2). These questions are answered on a 5-point Likert scale ranging from “agree completely” (1) to “do not agree at all” (5), there is also the option “not relevant” (6). The constructs could be summed and used as subscales, but the questions could also be considered as single questions. All the questions were worded in a positive way except for question 13 (FTI disrupts working relationships), which was negatively worded. This question was consequently reversed and recoded before analysis. In the present study, Cronbach's alpha's values for the four constructs were 0.46 for *coherence,* 0.79 for *cognitive participation*, 0.43 for *collective action*, and 0.75 for *reflexive monitoring*. As S-NoMAD is relatively new, and hardly used in the Swedish healthcare context (50), it is used in its original form with the result both from the constructs and single questions.

**Table 3:** Background characteristics of the hospital social workers (*n* = 21).

Baseline characteristics of hospital social workers	*N* = 21
Gender	
Male, *n* (%)	1 (4.8)
Female, *n* (%)	20 (92.5)
Age	
Mean (SD)	48 (11.8)
Min-Max	27–65
Licenced as a “Healthcare Counsellor”	
Yes, *n* (%)	9
No, *n* (%)	12
Number of years working as a HSW	
Mean (SD)	10 (6.9)
Min-Max	2–25
Unit, *n* (%)	
Paediatric care	7 (33.3)
Cancer care (adult care)	5 (33.8)
Specialised palliative homecare for adults	9 (42.9)

### Data analysis

Descriptive statistics were used to describe the sample's characteristics and study variables. Non-ordered categorical data were presented as frequencies and percentages, ordered categories as median (Mdn) and percentiles (P_25_–P_75_), and continuous data as means and standard deviations (SD). Since the study was based on repeated measures, only HSWs with complete data from all three time points were included. To minimize the risk of a biased sample, comparisons of the baseline characteristics were made between the HSWs who completed all three assessments and those who did not, using independent sample *t*-test and chi-square test.

Friedman's test was used to evaluate HSWs perceptions of the implementation of FTI (i.e., S-NoMAD). The tests were conducted both on the single questions and the constructs. Wilcoxon signed-rank test with Bonferroni corrected *p*-values were used as *post-hoc* test.

Due to the small sample size, and thereby an increased risk of type II errors, both Bonferroni corrected, and non-corrected results are presented. Kendall's W was used as an effect size measure: 0.1– < 0.3 small effect, 0.3 – < 0.5 moderate effect, and ≥0.5 large effect. The level of statistical significance was set at 5% (*p* < 0.05). All analyses were conducted in the SPSS (53).

## Results

### Participant characteristics

**Figure 2:**
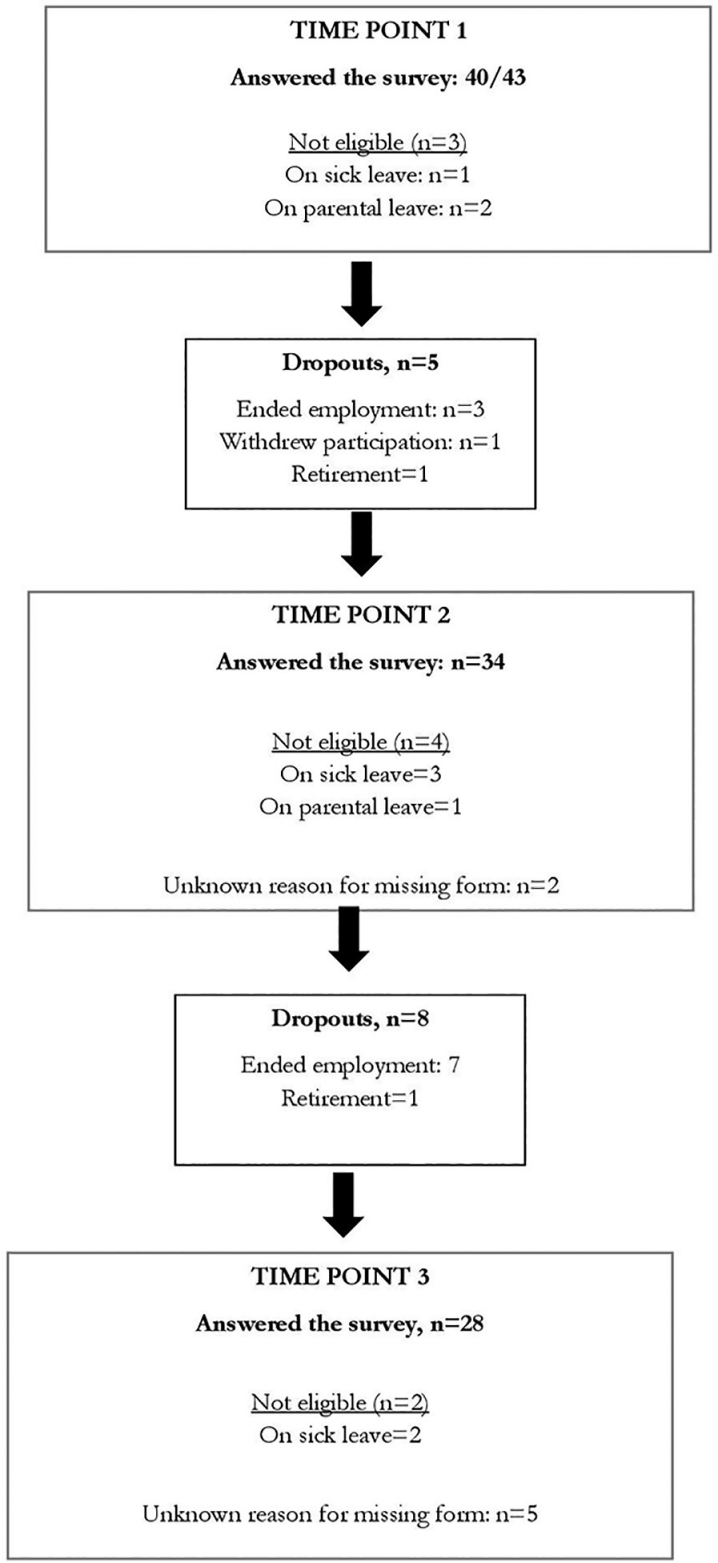
Flowchart for study subjects: answered at all time points *N* = 21.

In total, 21 HSWs completed S-NoMAD at all three time points (see Table 3). Their mean age was 48 years (min-max: 27–65 years) and all but one were women. They had worked as a HSW for an average of 10 years (min-max: 2–25 years). No significant differences were found between HSWs who completed all measurements (*n* = 21) and those who did not (*n* = 19). Reasons for dropping out of the study were mainly due to HSWs ending their employment; other reasons are illustrated in Figure 2. There were also several changes in management at some of the clinics during the research project.

### General questions

The results of Friedman's test regarding the three general questions in S-NoMAD demonstrated that for the HSWs, the FTI intervention became more familiar and normalized over time (Table 4). A significant change was found for the first general question in S-NoMAD where HSWs reported more familiarity with using FTI over time (*p* = 0.013). The uncorrected *post-hoc* tests demonstrated that the change was significant both between T1 and T3 and between T2 and T3; however, the significant difference between T2 and T3 was lost in the Bonferroni correction. A significant positive change over time was also seen regarding the HSWs reports about FTI as a normal part of their work (*p* = 0.025). Both corrected and uncorrected *post-hoc* tests showed that a significant change occurred between T2 and T3, where HSWs median score increased from 4 to 6 on a scale between 0 and 10. No significant change was observed for the question of whether HSWs believed that FTI *will* become a normal part of their work. However, HSWs rated the question highly from T1–T3 as the median score was stable and high, being 7 at all three time points.

**Table 4:** Descriptive statistics and the result of Friedman's test for the constructs and items of the S-NoMAD tool for participants (*n* = 21) with complete data.

Construct	Directly after^a^ FTI-education (T1)	Four months post FTI-education (T2)	One year post^b^ FTI-education (T3)	*P*-value^c^	Effect size^d^	Post hoc^e^ analyses, differences
General questions						
N1. When you use FTI how familiar does it feel?	6 (4–7, 3–10)	6 (5–8, 1–9)	8 (6–9, 3–10)	0.013	0.207	T1–T3* T2-T3
N2. Do you feel that FTI is currently a normal part of your work?	4 (4–5, 2–8)	5 (4–7, 1–9)	6 (5–7, 1–10)	0.025	0.194	T1–T3*
N3. Do you feel that FTI will become a normal part of your work?	7 (6–8, 3–10)	7 (5–9, 1–10)	7 (5–8, 1–10)	0.646	0.020	NS
Coherence	2 (2–2, 1–3)	2 (1–2, 1–3)	2 (1–2, 1–3)	0.374	0.052	NS
N4. I can see how FTI differs from usual ways of working	1 (1–2, 1–2)	1 (1–2, 1–2)	2 (1–2, 1–2)	0.307	0.059	NS
N5. Hospital social workers in this organisation have a shared understanding of the purpose of FTI	2 (1–2, 1–5)	2 (1–3, 1–4)	2 (1–3, 1–5)	0.315	0.024	NS
N6. I understand how FTI affects the nature of my own work	2 (2–2, 1–3)	2 (1–2, 1–3)	2 (1–2, 1–3)	0.620	0.0.24	NS
N7. I can see the potential value of FTI for my work.	1 (1–2, 1–2)	2 (1–2, 1–2)	1 (1–2, 1–2)	0.670	0.02	NS
Cognitive Participation	2 (1–2, 1–3)	2 (2–2, 1–3)	2 (1–2, 1–3)	0.215	0.006	NS
N.8 There are key people who drives FTI forward and get others involved	2 (1–2, 1–3)	2 (1–2, 1–4)	2 (1–2, 1–5)	0.667	0.020	NS
N9. I believe that participating in FTI is a legitimate part of my role	2 (1–2, 1–3)	2 (2–2, 1–4)	2 (1–2, 1–4)	0.982	0.001	NS
N10. I'm open to working with colleagues in new ways to use FTI	2 (1–2, 1–3)	2 (1–2, 1–3)	1 (1–2, 1–4)	0.620	0.024	NS
N11. I will continue to support FTI	2 (1–2, 1–3)	2 (1–2, 1–3)	1 (1–2, 1–3)	0.292	0.065	NS
Collective Action	2 (2–3, 1–3)	2 (2–3, 1–4)	2 (2–3, 3–3)	0.153	0.099	NS
N12. I can easily integrate the FTI into my existing work	3 (2–3, 1–5)	3 (3–4, 2–4)	3 (2–3, 1–5)	0.121	0.101	NS
N13. FTI disrupts working relationships	2 (1–2, 1–3)	2 (1–3, 1–3)	2 (1–3, 1–4)	0.382	0.064	NS
N14. I have confidence in other people's ability to use the FTI	2 (1–2, 1–3)	2 (1–2, 1–3)	2 (1–2, 1–3)	0.918	0.005	NS
N15. Work is assigned to those with skills appropriate to the FTI	2 (2–2, 1–3)	2 (2–2, 1–3)	2 (2–2, 1–5)	0.486	0.040	NS
N16. Sufficient training is provided to enable hospital social workers to use FTI	2 (1–2, 1–3)	2 (1–2, 1–3)	2 (2–3, 1–4)	0.052	0.141	NS
N17. Sufficient resources are available to support the FTI	2 (2–4, 1–5)	3 (2–4, 1–5)	3 (2–3, 1–5)	0.642	0.021	NS
N18. Management adequately support FTI	2 (2–3, 1–3)	2 (2–3, 1–3)	3 (2–3, 1–4)	0.294	0.061	NS
Reflexive monitoring	2 (2–2, 1–3)	2 (2–2, 1–3)	2 (2–2, 1–3)	0.933	0.005	NS
N19. I am aware of reports about the effects of the FTI	2 (2–2, 1–3)	2 (2–2, 1–3)	2 (2–2, 1–3)	0.171	0.004	NS
N20. Hospital social workers agree that the FTI is worthwhile	2 (2–3, 1–4)	2 (2–3, 1–4)	2 (2–3, 1–4)	0.358	0.057	NS
N21. I value the effects the FTI has had on my work	2 (1–2, 1–3)	2 (1–2, 1–4)	2 (1–2, 1–3)	0.832	0.009	NS
N22. Feedback about FTI can be used to improve it in the future	2 (1–2, 1–3)	2 (1–2, 1–2)	2 (2–2, 1–3)	0.199	0.095	NS
N23. I can modify how I work with FTI	2 (2–3, 1–3)	2 (1–3, 1–4)	2 (1–2, 1–5)	0.627	0.023	NS

^a^ Data are median (q1–q3; min-max).

^b^ Friedman's test.

^c^ Kendall's W Value: 0.1– < 0.3 (small effect), 0.3– < 0.5 (moderate effect) and ≥0.5 (large effect).

^d^ Significant differences from *post hoc* analysis (Wilcoxon signed-rank or McNemar test); * indicates significant differences following Bonferroni correction.

^e^ Lower score = better.

### Coherence

The ratings of the construct *coherence* in S-NoMAD were stable with a median score of 2 (agree) for all three time points, indicating that the HSWs attitude to *the differentiation, communal specification, individual specification, and internalization* of FTI was positive, with no significant changes. The median score across the questions in the construct (N4–N7) were 1 (agree completely) or 2 (agree) and did no change between the three time points.

The questions in the construct (N4–N7) ranged between 1 and 2 (agree completely- agree) for all questions at all three time points.

### Cognitive participation

A similar positive attitude was shown for *cognitive participation* (*initiation, enrolment, legitimation, and activation*) with median scores of 2 (agree) for all three time points.

### Collective action

The HSWs ratings of the construct *collective action* (*interactional workability, relational integration, skill-set workability, and contextual integration*) of FTI had ratings or median 2 (agree) at all three time points. However, in the individual questions (N12–N18) lower scores were given regarding their ability to integrate FTI into their daily work (N12), but also to the resources provided (N17), sufficient training (N16), and support from their management (N18). The median scores for these questions ranged between 2 and 3 (agree-neither agree nor disagree). Over the three time points, lower scores were given regarding provided resources (N17) and support from their managers (N18). A tendency for significant changes was seen over time for question N16, sufficient training (*p* = 0.052), indicating a greater variation in the ratings.

### Reflexive monitoring

HSW ratings for the construct *reflexive monitoring* (*systematization, communal appraisal, individual appraisal, reconfiguration*) were very stable with median scores of 2 (agree) for all time points. Similarly, median scores for all questions (N19–N23) at all time points were also 2.

## Discussion

HSWs were mainly positive in their initial ratings of using FTI in their everyday work, leaving little room for improvement over time. The result of S-NoMAD indicated that the multifaced implementation strategy facilitated the HSW implementation of FTI into their everyday clinical practice, from the beginning, but also over time to keep the work with FTI going. However, contextual factors related to the implementation of FTI were rated less favourably by the HSWs, such as management strategies and the resources allocated for FTI.

By using the S-NoMAD and thereby the NPT framework, this study offers important insights into the work that HSWs engage when integrating FTI into their routine work, based on their social and contextual factors (30, 50). Hence, the framework can provide a deeper understanding regarding how and why FTI was integrated from the perspective of HSWs (30). As there is no standardized way to coop with challenges that arise during an implementation process, this study can provide insights into which factors that affected the implementation and why (38). By understanding this core mechanism, which can either hinder or facilitate implementation, strategies to support the implementation process can be developed (28). As psychosocial intervention often is complex to implement, e.g., involving whole entire families, and addressing multiple needs (26, 31), this knowledge is of particular importance.

Significant positive changes were seen over time in the ratings of the two first general questions since FTI became more familiar and normalized over time, indicating that the implementation strategy also facilitated the HSWs continued efforts to integrate FTI into their daily work. The result adds to earlier research (54) showing the value of multifaceted implementation strategies in making new interventions in healthcare become routine (36, 55). Since the implementation strategy was dynamic, this enabled the external facilitators to mediate the expressed implementation support needs from the HSWs to the key facilitator. For example, since the HSWs reported that the recruitment of families to FTI was challenging (56), they were offered help from the facilitators with this. This is in line with previous research showing that facilitation can be used flexibly to manage problems that arise when implementing new interventions (57, 58).

In line with our findings, previous research has demonstrated that a positive attitude towards new interventions in healthcare facilitates their implementation (59). In the present study, the ratings of the constructs in S-NoMAD *coherence, cognitive participation,* and *reflexive monitoring* were generally positive and stable. However, the HSWs rated the construct *collective action* somewhat lower*,* referring to support from managers and the resources provided. These factors can be seen as part of the outer setting, which could influence the inner setting, for example, the implementation climate. The lower ratings for these outer setting factors could be due to management changes during the implementation process, which might have impacted the implementation of FTI. This corresponds with earlier implementation research, suggesting the importance of close managerial support when implementing new interventions in healthcare (60, 61).

Another question in the *collective action* construct rated more negatively over time was whether HSWs could easily integrate FTI into their work. As the HSW's operate within a medical context, it might have been challenging to implement FTI in a context not customized for psychosocial support. A potentially useful component in a multifaceted implementation strategy might have been internal (local) facilitators who better could have supported the HSWs influencing contextual barriers (33).

### Methodological considerations

The importance of implementing evidence-based ways of working into HSWs practice has been raised earlier (62, 63). This study has a unique strength as it involves the implementation process of a family-based psychosocial intervention delivered by HSWs among families living with severe illness, as earlier intervention studies have mainly focused on intervention effectiveness (14, 15). The current intervention, FTI, has been delivered in real-life conditions to vulnerable populations. The study includes a wide representation of HSWs with different working experience from various healthcare settings. In addition, since data were collected longitudinally over 16 months, more all-encompassing results can be presented from the implementation process than if the design had been cross-sectional. The used sample represented various working units, both adult and children's care, implying that the results from the study might be applicable to similar psychosocial interventions in several healthcare contexts.

Limitations of the study include its small sample size and the risk of type-II errors. The original inclusion of 43 HSWs was reduced to 21 in the final sample, mainly due to the HSWs changing workplace. A high level of job resignations was observed over time, contributing to the high attrition. This reflects the difficulties that arise when conducting implementation studies within clinical practice, where this kind of dropout can occur. The challenges of staff turnover during the implementation of new intervention has been raised before (64). Nevertheless, no differences regarding background characteristics were found between the initially included HSWs and the sample used for this study. As the study population is homogenous, e.g., similar educational background, a small sample size may be adequate (65). In longitudinal studies, sample sizes commonly decrease over time, with attrition being more common with long time intervals between the time points (65, 66). This reflects the difficulties that arise when conducting implementation studies within clinical practice, where this kind of dropout can occur. In longitudinal studies, sample sizes commonly decrease over time, with attrition being more common with long time intervals between the time points.

Implementation fidelity refers to what degree an intervention is delivered as it was intended (67), in this case to what degree the HSWs delivered FTI according to the intervention description. Within the current FTI-study, discussions were held between the external facilitators and HSWs regularly regarding how FTI should be delivered (56). Fidelity was also a central topic in the supervision sessions with the key-facilitator where adherence to the manual was discussed. Even if several strategies for fidelity were conducted in this study, we cannot present evidence that complete fidelity was the case. However, flexibility is often necessary to fit the specific context, and it does not mean that the fidelity of core components of the innovation is threatened (68).

The Cronbach´s alpha values for the constructs in S-NoMAD ranged from 0.43 to 0.79, indicating a varied internal consistency. This differs from the Elf, et al. (50) Swedish study (50) using S-NoMAD in health care among health care professionals (not HSWs), which showed higher alpha values. However, since the constructs include few questions, this could influence the test and result in lower alpha values, without necessarily meaning that the internal consistency is low (69). This sample is also relatively small, which might also be one reason for low alpha values on two constructs in S-NoMAD (*Coherence* and *Collective action*). The *Collective action* construct included a question that was negatively worded (FTI disrupts working relationship) unlike the other questions which could also incorporate lower internal consistency (70).

## Conclusion

The HSWs mainly rated different aspects of the implementation process as positive, from the beginning but also over time. FTI became significantly more familiar and normalized over time. The results indicate that the multifaced implementation strategy including e.g., education and facilitation used to support the implementation of FTI into everyday clinical practice could be considered successful. However, contextual factors such as managerial support and resources were rated lower, leaving opportunities for improvement. The lower rating within these constructs demonstrates the importance of considering contextual aspects when introducing interventions into healthcare. Based on our result, it is recommended that, prior to initiating the implementation process, the work environment, be assessed, e.g., by ensuring adequate resources, team involvement and identifying the type of managerial support required. This assessment can serve as an inventory to determine the context-specific support needed throughout the implementation process.

## Data Availability

The datasets presented in this article are not readily available because Related to individual privacy, the generated dataset and analytical data for the current study are not publicly available. Requests to access the datasets should be directed to malin.lovgren@mchs.se.

## References

[1] Caruso R Nanni MG Riba MB Sabato S Grassi L. The burden of psychosocial morbidity related to cancer: patient and family issues. Int Rev Psychiatry. (2017) 29(5):389–402. 10.1080/09540261.2017.1288090 28753076

[2] Kühne F Krattenmacher T Beierlein V Grimm JC Bergelt C Romer G Minor children of palliative patients: a systematic review of psychosocial family interventions. J Palliat Med. (2012) 15(8):931–45. 10.1089/jpm.2011.0380 22849598 PMC3396138

[3] Lin B Gutman T Hanson CS Ju A Manera K Butow P Communication during childhood cancer: systematic review of patient perspectives. Cancer. (2020) 126(4):701–16. 10.1002/cncr.32637 31821552

[4] Birgisdottir D Bylund Grenklo T Nyberg T Kreicbergs U Steineck G Furst CJ. Losing a parent to cancer as a teenager: family cohesion in childhood, teenage, and young adulthood as perceived by bereaved and non-bereaved youths. Psychooncology. (2019) 28(9):1845–53. 10.1002/pon.5163 31250504 PMC6771813

[5] Long KA Lehmann V Gerhardt CA Carpenter AL Marsland AL Alderfer MA. Psychosocial functioning and risk factors among siblings of children with cancer: an updated systematic review. Psychooncology. (2018) 27(6):1467–79. 10.1002/pon.4669 29441699

[6] Phillips F. Adolescents living with a parent with advanced cancer: a review of the literature. Psychooncology. (2014) 23(12):1323–39. 10.1002/pon.3570 24911540

[7] Dalton L Rapa E Ziebland S Rochat T Kelly B Hanington L Communication with children and adolescents about the diagnosis of a life-threatening condition in their parent. Lancet. (2019) 393(10176):1164–76. 10.1016/S0140-6736(18)33202-1 30894272

[8] Stein A Dalton L Rapa E Bluebond-Langner M Hanington L Stein KF Communication with children and adolescents about the diagnosis of their own life-threatening condition. Lancet. (2019) 393(10176):1150–63. 10.1016/S0140-6736(18)33201-X 30894271

[9] Bitschnau KW Firth P Wasner M. Social work in hospice and palliative care in Europe: findings from an EAPC survey. Palliat Support Care. (2020) 18(6):662–9. 10.1017/S1478951520000279 33399038

[10] Taels B Hermans K Van Audenhove C Boesten N Cohen J Hermans K How can social workers be meaningfully involved in palliative care? A scoping review on the prerequisites and how they can be realised in practice. Palliat Care Soc Pract. (2021) 15:26323524211058895. 10.1177/26323524211058895 34870204 PMC8637690

[11] Gomes-Ferraz CA Rezende G Fagundes AA De Carlo M. Assessment of total pain in people in oncologic palliative care: integrative literature review. Palliat Care Soc Pract. (2022) 16:26323524221125244. 10.1177/26323524221125244 36172038 PMC9511321

[12] IFSW. Global definition of social work. Available at: https://www.ifsw.org/what-is-social-work/global-definition-of-social-work/2014

[13] Lundin A Benkel I Neergaard GD Johansson B-M Öhrling C. Kurator Inom Hälso- och Sjukvård. Lund: Studentlitteratur (2019).

[14] Ellis SJ Wakefield CE Antill G Burns M Patterson P. Supporting children facing a parent’s cancer diagnosis: a systematic review of children’s psychosocial needs and existing interventions. Eur J Cancer Care. (2017) 26(1):e12432. 10.1111/ecc.12432 26776913

[15] Steiner V Shlonsky A Joubert L. Psychosocial interventions for parents with incurable end-stage cancer: a rapid evidence assessment. Aust Psychol. (2017) 52(5):381–91. 10.1111/ap.12286

[16] Henoch I Carlander I Holm M James I Sarenmalm EK Hagelin CL Palliative care research–a systematic review of foci, designs and methods of research conducted in Sweden between 2007 and 2012. Scand J Caring Sci. (2016) 30(1):5–25. 10.1111/scs.12253 26190052

[17] Barth RP Lee BR Lindsey MA Collins KS Strieder F Chorpita BF Evidence-based practice at a crossroads. Res Soc Work Pract. (2011) 22(1):108–19. 10.1177/1049731511408440

[18] Wike TL Bledsoe SE Manuel JI Despard M Johnson LV Bellamy JL Evidence-based practice in social work: challenges and opportunities for clinicians and organizations. Clin Soc Work J. (2014) 42(2):161–70. 10.1007/s10615-014-0492-3

[19] Udo C Forsman H Jensfelt M Flink M. Research use and evidence-based practice among Swedish medical social workers: a qualitative study. Clin Soc Work J. (2019) 47(3):258–65. 10.1007/s10615-018-0653-x

[20] Inhestern L Haller AC Wlodarczyk O Bergelt C. Psychosocial interventions for families with parental cancer and barriers and facilitators to implementation and use—a systematic review. PLoS One. (2016) 11(6):e0156967. 10.1371/journal.pone.0156967 27276079 PMC4898703

[21] Koumarianou A Symeonidi AE Kattamis A Linardatou K Chrousos GP Darviri C. A review of psychosocial interventions targeting families of children with cancer. Palliat Support Care. (2021) 19(1):103–18. 10.1017/S1478951520000449 32613930

[22] Alvariza A Jalmsell L Eklund R Lovgren M Kreicbergs U. The family talk intervention in palliative home care when a parent with dependent children has a life-threatening illness: a feasibility study from parents’ perspectives. Palliat Support Care. (2021) 19(2):154–60. 10.1017/S1478951520000735 32854809

[23] Eklund R Jalmsell L Kreicbergs U Alvariza A Lovgren M. Children’s experiences of the family talk intervention when a parent is cared for in palliative home care-A feasibility study. Death Stud. (2022) 46(7):1655–66. 10.1080/07481187.2020.1829747 33054633

[24] Eklund R Alvariza A Kreicbergs U Jalmsell L Lovgren M. The family talk intervention for families when a parent is cared for in palliative care—potential effects from minor children’s perspectives. BMC Palliat Care. (2020) 19(1):50. 10.1186/s12904-020-00551-y 32299420 PMC7164202

[25] Eklund R Lovgren M. The family talk intervention in pediatric oncology: ill Children’s descriptions of feasibility and potential effects. J Pediatr Hematol Oncol Nurs. (2022) 39(3):143–54. 10.1177/27527530221068423 35467434

[26] Moore G Audrey S Barker M Bond L Bonell C Cooper C Process evaluation in complex public health intervention studies: the need for guidance. J Epidemiol Community Health. (2014) 68(2):101–2. 10.1136/jech-2013-202869 24022816 PMC3892708

[27] Nilsen P. Making sense of implementation theories, models and frameworks. Implement Sci. (2015) 10:53. 10.1186/s13012-015-0242-0 25895742 PMC4406164

[28] May CR Cummings A Girling M Bracher M Mair FS May CM Using normalization process theory in feasibility studies and process evaluations of complex healthcare interventions: a systematic review. Implement Sci. (2018) 13(1):80. 10.1186/s13012-018-0758-1 29879986 PMC5992634

[29] Murray E Treweek S Pope C MacFarlane A Ballini L Dowrick C Normalisation process theory: a framework for developing, evaluating and implementing complex interventions. BMC Med. (2010) 8:63. 10.1186/1741-7015-8-63 20961442 PMC2978112

[30] Dalkin SM Hardwick RJL Haighton CA Finch TL. Combining realist approaches and normalization process theory to understand implementation: a systematic review. Implement Sci Commun. (2021) 2(1):68. 10.1186/s43058-021-00172-3 34174966 PMC8234627

[31] Skivington K Matthews L Simpson SA Craig P Baird J Blazeby JM A new framework for developing and evaluating complex interventions: update of medical research council guidance. Br Med J. (2021) 374:n2061. 10.1136/bmj.n2061 34593508 PMC8482308

[32] Wolfenden L Foy R Presseau J Grimshaw JM Ivers NM Powell BJ Designing and undertaking randomised implementation trials: guide for researchers. Br Med J. (2021) 372:m3721. 10.1136/bmj.m3721 33461967 PMC7812444

[33] Harvey G Kitson A. PARIHS Revisited: from heuristic to integrated framework for the successful implementation of knowledge into practice. Implement Sci. (2016) 11:33. 10.1186/s13012-016-0398-2 27013464 PMC4807546

[34] Pinnock H Barwick M Carpenter CR Eldridge S Grandes G Griffiths CJ Standards for reporting implementation studies (StaRI): explanation and elaboration document. BMJ Open. (2017) 7(4):e013318. 10.1136/bmjopen-2016-013318 28373250 PMC5387970

[35] Proctor EK Powell BJ McMillen JC. Implementation strategies: recommendations for specifying and reporting. Implement Sci. (2013) 8:139. 10.1186/1748-5908-8-139 24289295 PMC3882890

[36] Powell BJ Waltz TJ Chinman MJ Damschroder LJ Smith JL Matthieu MM A refined compilation of implementation strategies: results from the expert recommendations for implementing change (ERIC) project. Implement Sci. (2015) 10:21. 10.1186/s13012-015-0209-1 25889199 PMC4328074

[37] Mills K Griffin SJ Sutton S Usher-Smith JA. Development and usability testing of a very brief intervention for personalised cancer risk assessment to promote behaviour change in primary care using normalisation process theory. Prim Health Care Res Dev. (2020) 21:e1. 10.1017/S146342361900080X 31934843 PMC7005588

[38] Lynch EA Mudge A Knowles S Kitson AL Hunter SC Harvey G. There is nothing so practical as a good theory": a pragmatic guide for selecting theoretical approaches for implementation projects. BMC Health Serv Res. (2018) 18(1):857. 10.1186/s12913-018-3671-z 30428882 PMC6236961

[39] Curran GM Landes SJ McBain SA Pyne JM Smith JD Fernandez ME Reflections on 10 years of effectiveness-implementation hybrid studies. Front Health Serv. (2022):2.1053496. 10.3389/frhs.2022.1053496 36925811 PMC10012680

[40] Beardslee WR Swatling S Hoke L Rothberg PC van de Velde P Focht L From cognitive information to shared meaning: healing principles in prevention intervention. Psychiatry. (1998) 61(2):112–29. 10.1080/00332747.1998.11024822 9706099

[41] Pihkala H Sandlund M Cederström A. Children in Beardslee's family intervention: relieved by understanding of parental mental illness. Int J Soc Psychiatry. (2012) 58(6):623–8. 10.1177/0020764011419055 21900288

[42] Beardslee WR. When a parent is depressed: how to protect your children from the effects of depression in the family. (2002).

[43] Eklund R Kreicbergs U Alvariza A Lovgren M. Children’s views are not taken into account in accordance with article 12 of the united nations convention on the rights of the child in the family talk intervention when a parent is cared for in palliative care. Omega (Westport). (2022) 85(1):126–54. 10.1177/0030222820941283 32659170

[44] Solantaus T Paavonen EJ Toikka S Punamaki RL. Preventive interventions in families with parental depression: children’s psychosocial symptoms and prosocial behaviour. Eur Child Adolesc Psychiatry. (2010) 19(12):883–92. 10.1007/s00787-010-0135-3 20890622 PMC2988995

[45] Ayoub M Udo C Arestedt K Kreicbergs U Lovgren M. The family talk intervention in pediatric oncology: potential effects reported by parents. Children. (2024) 11(1):95. 10.3390/children11010095 38255408 PMC10814711

[46] Harvey G Kitson A. Implementing Evidence-Based Practice in Healthcare: A Facilitation Guide. London: Routledge/Taylor & Francis Group (2015).

[47] WHO. Palliative Care. WHO (2020). Available at: https://www.who.int/news-room/fact-sheets/detail/palliative-care#:∼:text=Palliative%20care%20medicines%2C%20including%20those%20for%20pain%20relief%2C,noncommunicable%20diseases%2C%20and%20people-centred%20and%20integrated%20health%20services (Accessed May 20, 2024).

[48] Benini F Papadatou D Bernada M Craig F De Zen L Downing J International standards for pediatric palliative care: from IMPaCCT to GO-PPaCS. J Pain Symptom Manage. (2022) 63(5):e529–e43. 10.1016/j.jpainsymman.2021.12.031 35031506

[49] World Medical Association. World medical association declaration of Helsinki: ethical principles for medical research involving human subjects. JAMA. (2013) 310(20):2191–4. 10.1001/jama.2013.281053 24141714

[50] Elf M Nordmark S Lyhagen J Lindberg I Finch T Aberg AC. The Swedish version of the normalization process theory measure S-NoMAD: translation, adaptation, and pilot testing. Implement Sci. (2018) 13(1):146. 10.1186/s13012-018-0835-5 30509289 PMC6278165

[51] May CR Mair F Finch T MacFarlane A Dowrick C Treweek S Development of a theory of implementation and integration: normalization process theory. Implement Sci. (2009) 4:29. 10.1186/1748-5908-4-29 19460163 PMC2693517

[52] May C Rapley T Mair FS Treweek S Murray E Ballini L Normalization Process Theory On-line Users’ Manual, Toolkit and NoMAD instrument. (2015) Available at: http://www.normalizationprocess.org (Accessed August 20, 2025).

[53] Corp I. IBM SPSS Statistics for Windows. NY: Armonk (2020).

[54] Damschroder LJ Reardon CM Widerquist MAO Lowery J. The updated consolidated framework for implementation research based on user feedback. Implement Sci. (2022) 17(1):75. 10.1186/s13012-022-01245-0 36309746 PMC9617234

[55] Cassidy CE Harrison MB Godfrey C Nincic V Khan PA Oakley P Use and effects of implementation strategies for practice guidelines in nursing: a systematic review. Implement Sci. (2021) 16(1):102. 10.1186/s13012-021-01165-5 34863220 PMC8642950

[56] Thermaenius I Udo C Alvariza A Lundberg T Holm M Lovgren M. The family talk intervention among families affected by severe illness: hospital social Workers’ experiences of facilitators and barriers to its use in clinical practice. J Soc Work End Life Palliat Care. (2024) 20:235–53. 10.1080/15524256.2024.2364589 38968160

[57] Bidassie B Williams LS Woodward-Hagg H Matthias MS Damush TM. Key components of external facilitation in an acute stroke quality improvement collaborative in the veterans health administration. Implement Sci. (2015) 10:69. 10.1186/s13012-015-0252-y 25971405 PMC4437451

[58] Stetler CB Legro MW Rycroft-Malone J Bowman C Curran G Guihan M Role of “external facilitation” in implementation of research findings: a qualitative evaluation of facilitation experiences in the veterans health administration. Implement Sci. (2006) 1:23. 10.1186/1748-5908-1-23 17049080 PMC1635058

[59] Fischer F Lange K Klose K Greiner W Kraemer A. Barriers and strategies in guideline implementation-A scoping review. Healthcare. (2016) 4(3):36. 10.3390/healthcare4030036 27417624 PMC5041037

[60] Gifford W Graham ID Ehrhart MG Davies BL Aarons GA. Ottawa model of implementation leadership and implementation leadership scale: mapping concepts for developing and evaluating theory-based leadership interventions. J Healthc Leadersh. (2017) 9:15–23. 10.2147/JHL.S125558 29355212 PMC5774448

[61] Zjadewicz K White D Bouchal SR Reilly S. Middle managers’ role in quality improvement project implementation, are we all on the same page?—a review of current literature. Safety Health. (2016) 2(1):1–7. 10.1186/s40886-016-0018-5

[62] Ståhl D Lundälv J. Health social workers and research knowledge utilisation—a Swedish survey study. Eur J Soc Work. (2022) 26:908–21. 10.1080/13691457.2022.2148092

[63] Ståhl D Lundälv J. Research knowledge utilisation among Swedish health social workers: influence of higher education, job tenure and workplace. Int Soc Work. (2024) 67:1211–25. 10.1177/00208728241235264

[64] Aarons GA Sommerfeld DH Hecht DB Silovsky JF Chaffin MJ. The impact of evidence-based practice implementation and fidelity monitoring on staff turnover: evidence for a protective effect. J Consult Clin Psychol. (2009) 77(2):270–80. 10.1037/a0013223 19309186 PMC2742697

[65] Polit DF Beck CT. Nursing Research: Generating and Assessing Evidence for Nursing Practice. Philadelphia: Wolters Kluwer (2021).

[66] Twisk J de Vente W. Attrition in longitudinal studies. How to deal with missing data. J Clin Epidemiol. (2002) 55(4):329–37. 10.1016/S0895-4356(01)00476-0 11927199

[67] Damschroder LJ Reardon CM Opra Widerquist MA Lowery J. Conceptualizing outcomes for use with the consolidated framework for implementation research (CFIR): the CFIR outcomes addendum. Implement Sci. (2022) 17(1):7. 10.1186/s13012-021-01181-5 35065675 PMC8783408

[68] Harn B Parisi D Stoolmiller M. Balancing fidelity with flexibility and fit: what do we really know about fidelity of implementation in schools? Except Child. (2013) 79(3):181–93. 10.1177/0014402913079002051

[69] Tavakol M Dennick R. Making sense of Cronbach’s alpha. Int J Med Educ. (2011) 2:53–5. 10.5116/ijme.4dfb.8dfd 28029643 PMC4205511

[70] Solis Salazar M. The dilemma of combining positive and negative items in scales. Psicothema. (2015) 27(2):192–200. 10.7334/psicothema2014.266 25927700

